# Optimal Staffing for Vessel Traffic Service Operators: A Case Study of Yeosu VTS

**DOI:** 10.3390/s21238004

**Published:** 2021-11-30

**Authors:** Sang-Lok Yoo, Kwang-Il Kim

**Affiliations:** 1 Future Ocean Information Technology, Jeju 63208, Korea; sanglokyoo@gmail.com; 2 Department of Marine Industry and Maritime Police, Jeju National University, Jeju 64343, Korea

**Keywords:** staffing level, workload, vessel traffic service, operator, automatic identification system

## Abstract

Vessel traffic volume and vessel traffic service (VTS) operator workloads are increasing with the expansion of global maritime trade, contributing to marine accidents by causing difficulties in providing timely services. Therefore, it is essential to have sufficient VTS operators considering the vessel traffic volume and near-miss cases. However, no quantitative method for determining the optimal number of workstations, which is necessary for calculating the VTS operator staffing level, has yet been proposed. This paper proposes a new, microscopic approach for calculating the number of workstations from vessel trajectories and voice recording communication data between VTS operators and navigators. The vessel trajectory data are preprocessed to interpolate different intervals. The proposed method consists of three modules: Information services, navigational assistance services, and traffic organization service. The developed model was applied to the Yeosu VTS in Korea. Another workstation should be added to the current workstation based on the proposed method. The results showed that even without annual statistical data, a reasonable VTS operator staffing level could be calculated. The proposed approach helps prevent vessel accidents by providing timely services even if the vessel traffic is congested if VTS operators are deployed to a sufficient number of workstations.

## 1. Introduction

With the rapid growth of global trade and maritime transportation, vessel traffic services (VTS) have come to play a critical role in guaranteeing maritime safety, facilitating traffic flow, and safeguarding the environment. VTS is an integrated shore-side service provided by competent authorities that includes a variety of navigational assistance for vessels and extensive traffic control in a designated maritime area. The VTS operators collect information about the geographical area they monitor using a variety of sensors. A VTS operator normally uses automatic identification systems (AIS), radar, closed-circuit television (CCTV), very-high-frequency (VHF) marine radio, and pilot scheduled data to monitor the movements of vessels in a VTS area.

VTS operators work in shifts and have irregular hours. The VTS operators work in a 24-h shift, which causes fatigue. Moreover, the number of vessels in the VTS area varies daily. Therefore, VTS operators should adapt quickly to new situations daily. In addition, they must maintain intense concentration and deal with critical situations and emergencies. As a result, VTS operators will undoubtedly experience fatigue. Additionally, it is well established that fatigue on the part of VTS operators can pose a catastrophic risk to human life and damage the environment and property [1]. A staffing level that is only capable of handling the expected workload will undoubtedly be overloaded when an unexpected event, emergency, or incident occurs.

There is a lack of staffing among VTS operators because of the large number of vessels [2]. The number of VTS operators on duty at any given time is determined by the safe and efficient operation of the VTS area and is reflected in human resource planning, including staff rotation and rest period arrangements within any given shift or watch. Moreover, VTS operators operate in a state where a considerable burden is inherent in their work to ensure that vessels can navigate safely. VTS centers are even expanding their monitoring areas of responsibility while maintaining current staffing levels, resulting in an increase in the workload of operators [3]. Hyperstress may prevent personnel from effectively interpreting data, making sound judgments, and taking appropriate actions. Numerous maritime accidents occur in the VTS area [4]. According to the Korea Maritime Safety Tribunal statistics, a total of 216 vessel collision accidents occurred in ports over the past five years (2016–2020), and a total of 879 ship collision accidents occurred in territorial waters [5]. In addition, 15 VTS centers are operated nationwide in Korean ports, and 19.7% of all collision accidents occur in the VTS area. In some cases, VTS operators have failed to meet their responsibilities [6,7]. In particular, the sunken ferry Sewol accident was a VTS operator missing early detection. The VTS operators ignored the service regulation that two operators should be at work simultaneously; one has to monitor the coastal sea and the other to monitor the open sea [8,9]. Meanwhile, it was found that variables such as short and irregular meals and work overload due to lack of VTS operator staffing were correlated with job stress [10]. In other words, the staff level at the VTS center is critical.

The International Association of Marine Aids to Navigation and Lighthouse Authorities (IALA) oversees maintaining marine aids to navigation worldwide including VTS by issuing recommendations and guidelines to the member states. The IALA has developed a recommendation for determining the appropriate staffing level for a VTS (i.e., IALA guideline 1045) [11]. The purpose of the guideline is to assist authorities in determining the appropriate staffing level for a VTS center. It covers several variables that affect the workload of the VTS center. However, the guideline does not suggest calculating the correct number of workstations even though VTS staffing should be proportional to the number of workstations.

This paper presents a new vessel traffic flow-based optimal VTS operator staffing level model using AIS sensor data and VHF radio voice data. There are three modules, namely information services (INS), navigational assistance services (NAS), and traffic organization services (TOS), in the model. Each module is used to calculate the time needed for INS, NAS, and TOS. In addition, each module consists of several sub-items. The time needed for each module was calculated by adding the time required for each sub-item. In addition, the number of occurrences of each sub-item was calculated using the AIS sensor data. It introduces a preprocessing method for AIS sensor data to handle different asynchronous intervals and missing messages. The time needed for sub-items was derived through a questionnaire survey of VTS operators and VHF radio voice analysis. The required number of VTS workstations per hour can be calculated by adding the time required for the three modules. Finally, we provide recommendations to authorities to determine the optimal staff level to improve the service provided by the VTS center.

The remainder of this paper is organized as follows. Section 2 presents previous studies related to VTS operators. Section 3 describes how to preprocess the AIS sensor data and the optimal staffing level method of VTS operators. Section 4 presents the experimental results of the proposed method for a VTS center. Section 5 discusses the results in the context of the aims of the study. Finally, Section 6 concludes the study.

## 2. Related Works

Various studies have analyzed the workload and stress of VTS operators. It is reported that stress can be beneficial for emergencies, but excessive stress can have several negative effects. Therefore, the Maritime Port Authority rotates the positions of VTS operators in the Dover VTS and Singapore VTS, which are the busiest VTS, every 45 min [3]. In a previous study, Kum et al. [12] analyzed the relationship between the mental workload and variables, such as age, marital status, sea experience, VTS experience, and educational level, by conducting a questionnaire survey. However, they did not consider important factors, such as the number of vessels and course in the VTS area, limiting this study. In contrast, Xu et al. [13] proposed an adaptive rotating shift planning solution, which considers dynamic workload variables such as the number of vessels and speed, to prevent VTS operators from becoming tired. However, this method did not present a model for optimal staffing of VTS operators and did not focus on the number of VTS workstations required, concentrating only on the work shifts of VTS operators through a fixed number of VTS operators.

Moreover, fatigue has been reported to trigger various types of human errors [14]. Some studies have focused on the human errors of VTS operators. Kotkowska et al. [15] identified the errors committed by VTS operators by examining human nature-related factors, such as a lack of experience or qualifications, ineffective communication, fatigue, and routine. Various researchers have concluded that VTS operators are prone to errors as an inherent characteristic of human nature. Therefore, appropriate measures, such as adding VTS personnel, should be conducted to ensure that the required performance is maintained.

In recent years, numerous studies have been conducted to assist the decision-making abilities of VTS operators in reducing their workload and human errors. For example, Kim et al. [16] proposed a context-aware information provision model by applying deep learning and developed a decision support tool to predict vessel destinations. Moreover, Mazaheri et al. [17] developed a decision support tool to detect grounding candidate vessels in a VTS area. Meanwhile, Schuett [3] developed a system to reduce the workload of VTS operators by sending automated information such as weather data and vessel conflicts. In addition, methods for risk-based collision avoidance for VTS have been developed [18,19]. These computerized systems and decision tools can reduce the workload and stress for VTS operators. However, it is essential to deploy suitable staffing for an appropriate number of workstations even though a decision tool for VTS operators is already available.

A few studies have focused on performance and training programs for VTS operators [20,21]. It is clear that sound operating procedures, reliable decision tools, and operating personnel are necessary considerations. Xie [1] presented the appropriate staffing model of VTS operators considering the number of workstations based on the IALA guide 1045. The approach is a macroscopic model that calculates the number of workstations using annual incoming and outgoing vessel statistics. However, the number of near-miss occurrences, such as crossing and overtaking, cannot be extracted using this model. In addition, the reliability of this approach is low because annual statistics are values for one year accumulated manually rather than by an automated system.

Meanwhile, macroscopic and microscopic models estimate the capacity of the sector for air traffic controllers (ATC). The macroscopic model considers the geometry of the air traffic service sector and the direction of the air traffic flow [22]. On the other hand, microscopic models consider each aircraft trajectory with detailed information about the gate occupancy and taxiway routing. The microscopic model is more precise although they can be computationally intensive [23]. The vessel flow is determined by the microscopic vessel behavior, which is determined by the different factors of each position, speed, and course of the vessel [24]. Vessel near-misses, which are considered a burden for VTS operators, occur daily in the VTS area [25,26]. The microscopic approach reflecting a vessel’s near-miss risk analysis to calculate staffing is more realistic than the macroscopic approach. In this study, in contrast to the macroscopic scheme [1], our proposed scheme is a microscopic approach to determine the optimal number of VTS operators.

## 3. Proposed Optimal VTS Operators Staffing Method

### 3.1. VTS Responsibilities

The primary responsibilities of VTS include the assistance for safe navigation in VTS areas and pollution mitigation. These responsibilities are accomplished by providing three distinct types of services: INS, NAS, and TOS [27]. VTS operators need facilities to monitor the operational situation related to all maritime activities in a VTS area. In addition, VTS operators continuously observe using multiple screens while remaining seated in the same position. Subsequently, they deliver timely, relevant, and accurate information to communicate with vessels using VHF radio. Therefore, the time needed for the task of VTS operators $T_{task}$ is expressed as

(1) $$T_{task}=T_{INS}+T_{NAS}+T_{TOS}$$

where $T_{INS}$, $T_{NAS}$, and $T_{TOS}$, is the time needed for information services, navigational assistance services, and traffic organization services, respectively.

**Figure 1:**
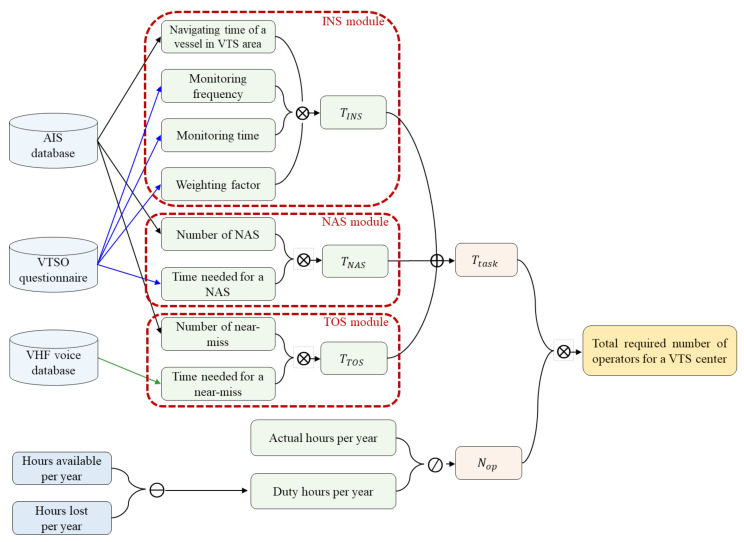
Optimal VTS staffing model process flow.

Figure 1 shows the proposed VTS optimal staffing-level calculation procedure, which included modules for computing the time needed for the INS, NAS, and TOS to calculate the time needed for each service. The number of occurrences for each module was extracted using the AIS data. In addition, the time needed for each module was calculated by adding the time needed for the sub-items composing each module, which was obtained through the questionnaire survey of VTS operators and voice analysis of VHF. Subsequently, the required number of workstations was calculated. The total required number of operators for a VTS center was obtained by multiplying the number of workstations needed and the number of VTS operators per workstation.

The number of VTS operators $N_{op}$ per workstation, which is the actual number of hours per year divided by duty hours per year, was calculated based on the IALA guidelines [11]. The actual hours per year are calculated as hours per day (i.e., 24) multiplied by the actual number of days per year (i.e., 365.25). On the other hand, the duty hours per year are calculated as hours available per year minus the hours lost per year, which includes hours of leave, sickness, and training per year.

### 3.2. Preprocessing of AIS Sensor Data

**Figure 2:**
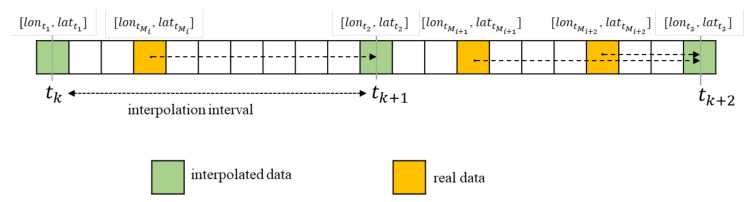
Real positions and the interpolated position.

AIS messages received at the VTS are sometimes missing, and their data intervals are different. Therefore, all data should be synchronized and interpolated at specific time intervals to analyze vessel near-miss. First, duplicated AIS messages sent by the same vessel were eliminated, and the position of the vessel at the reference time was determined using an interpolation method. Figure 2 shows the real data, including longitude, latitude, speed, and course, at irregular time intervals and interpolated data, including longitude, latitude, speed, and course, at a specific time interval. In the figure, the reference interval is the sampling period, $t_{k}$ is the *k*th reference time, and $t_{M_{i}}$ is the time of the *i*th real message $M_{i}$. Furthermore, the real position [$lon_{t_{M_{i}}}$, $lat_{t_{M_{i}}}$] is replaced with the position [$lon_{t_{k+1}}$, $lat_{t_{k+1}}$] at time $t_{k+1}$ if an AIS message $M_{i}$ occurs in the interval between the *k*th and (*k* + 1)th reference times (i.e., $t_{k}$ < $t_{M_{i}}$ < $t_{k+1}$).

The interpolated positions [$lon_{t_{k+1}}$, $lat_{t_{k+1}}$] at the (*k* + 1)th reference time are computed based on the vessel speed v and course θ from the current position [$lon_{t_{M_{i}}},\;lat_{t_{M_{i}}}$], as shown in Equation (2) [28].

(2) $$\left\{ \begin{array}{c} lat_{t_{k+1}}=\arcsin\left[\sin\left(lat_{t_{M_{i}}}\right)\cos\left(\frac{v\Delta t}{R}\right)+\cos\left(lat_{t_{M_{i}}}\right)\sin\left(\frac{v\Delta t}{R}\right)\cos\left(\theta \right)\right]\\ lon_{t_{k+1}}=lon_{t_{M_{i}}}+\arctan\left[\frac{\sin\left(\theta \right)\sin\left(\frac{v\Delta t}{R}\right)\cos\left(lat_{t_{M_{i}}}\right)}{\cos\left(\frac{v\Delta t}{R}\right)-\sin\left(lat_{t_{M_{i}}}\right)\sin\left(lat_{t_{k+1}}\right)}\right] \end{array}\right.$$

where Δt is the time between the *i*th real message $t_{M_{i}}$ and the (*k* + 1)th reference time $t_{k+1}$, and *R* is the radius of the earth.

### 3.3. Time Needed for INS

The task of VTS operators is to monitor hundreds of vessels simultaneously using several screens, which is possible due to the VTS system configuration. For example, VTS operators observe a VTS area by watching a screen, moving from the left to the lower right corner of the screen. Subsequently, after monitoring one screen, VTS operators turn right and continue the process with the following screens [29]. During the monitoring task, the VTS operators collect traffic data using integrated radar and AIS data. Knowing what to look for when monitoring is crucial. Therefore, monitoring is a prerequisite for INS [30,31]. The appropriate monitoring frequency varies across VTS centers and is determined by various criteria. For example, the higher-risk section necessitates more frequent monitoring [32].

$T_{INS}$ considers the navigating time of a vessel in a VTS area, monitoring frequency, monitoring time, and monitoring weighting factor based on the vessel type and length, as shown in Equation (3). This is a different importance factor by a section in a VTS area. For example, a precautionary section is where vessels must navigate cautiously. Moreover, VTS operators pay more attention to precautionary sections in which route intersections or traffic congestion sections are compared to other sections [33,34]. Therefore, the monitoring frequency and time were considered differently based on the section in a VTS area. Additionally, VTS operators pay more attention to high-risk vessels such as liquefied petroleum gas (LPG) tankers, liquefied natural gas (LNG) tankers, and very large crude carriers (VLCCs) compared to small vessels [35,36,37]. Therefore, the weighting factor is based on the vessel type and length for the time needed for monitoring.

(3) $$T_{INS}=\overset{S}{\underset{s=1}{\sum }}\overset{M}{\underset{m=1}{\sum }}\frac{n_{m}^{s}\;t^{s}\;w_{m}}{f^{s}}$$

where $n_{m}^{s}$ is the navigating time of the *m*th vessel at the *s*th section in a VTS area, consisting of a precautionary section, a fairway, an anchorage, a narrow channel, and open sea, $f^{s}$ and $t^{s}$ are the monitoring frequency and time at the *s*th section, respectively, and $w_{m}$ is the monitoring weighting factor based on the vessel type and length for the *m*th vessel.

### 3.4. Time Needed for NAS

NAS actively supports the onboard navigational decision-making process to assist vessels traversing in a VTS area. It consists of communication related to vessel movements, such as entering and leaving the VTS area, anchoring or berthing, heaving up anchor or unberthing, and pilot on board [38]. If a vessel moves in the VTS area, the VTS operator needs the primary time of communicating with vessels by VHF and has to note it in the VTS logbook. Additionally, depending on the vessel’s movement, time may be required to check the route and destination of the vessel, pilot scheduling, etc. [16,39]. Therefore, $T_{NAS}$ is added by the time required for each sub-item, as shown in Equation (4).

(4) $$T_{NAS}=\overset{E}{\underset{e=1}{\sum }}t_{e}+\overset{L}{\underset{l=1}{\sum }}t_{l}+\overset{A}{\underset{a=1}{\sum }}t_{a}+\overset{H}{\underset{h=1}{\sum }}t_{h}+\overset{P}{\underset{p=1}{\sum }}t_{p}$$

where $t_{e},\;t_{l},\;t_{a},\;t_{h},$ and $t_{p}$ are the times needed to communicate with the *e*th entering vessel in the VTS area, the *l*th leaving vessel, the *a*th anchoring or berthing vessel, the *h*th heaving up anchor vessel or unberthing, and the *p*th vessel with a pilot on board, respectively. Moreover, *E*, *L*, *A*, *H*, and *P* are the number of entering vessels, leaving vessels, anchoring or berthing vessels, heaving up anchor or unberthing vessels, and vessels with a pilot on board, respectively.

### 3.5. Time Needed for TOS

TOS is designed to prevent dangerous maritime traffic situations and ensure safe and efficient vessel traffic movements in a VTS area. Therefore, vessel movements must be planned or prioritized by VTS operators to avoid near-miss cases. In addition, TOS is performed by instructing or exercising the authority to direct movement.

The VTS operators analyze the near-miss based on the closest point of approach (CPA) of the vessel, which is an estimated location where the distance between vessels *A* and *B* is the shortest. Generally, the VTS operators determine the most appropriate collision avoidance action based on the distance at the closest point of approach (DCPA), which is the distance between vessels in close proximity, and the time to the closest point of approach (TCPA), which is the amount of time remaining until the two vessels reach their closest points. Therefore, the DCPA and TCPA are used as values to discriminate near-misses. If they are lower than the threshold, the VTS operator should provide a TOS regarding a near-miss situation. In this study, the threshold value of DCPA and TCPA was set to 0.5 nm and 5 min, respectively, according to the literature [40]. The DCPA and TCPA, which are expressed in Equations (5) and (6) [41], are calculated using interpolated data, including the longitude, latitude, speed, and course, of each vessel at the synchronous time.

(5) $$\mathrm{DCPA}=\sqrt{\begin{array}{c} {}[\Delta y+\left(v_{B}\sin\theta_{B}-v_{A}\sin\theta_{A}\right)\times \mathrm{TCPA}{]}^{2}+\\ {}[\Delta x+\left(v_{B}\cos\theta_{B}-v_{A}\cos\theta_{A}\right)\times \mathrm{TCPA}{]}^{2} \end{array}}$$

(6) $$\mathrm{TCPA}=-\frac{\left[\Delta y\;\left(v_{B}\sin\theta_{B}-v_{A}\sin\theta_{A}\right)+\Delta x\;\left(v_{B}\cos\theta_{B}-v_{A}\cos\theta_{A}\right)\right]}{{\left(v_{B}\sin\theta_{B}-v_{A}\sin\theta_{A}\right)}^{2}\;–\;{\left(v_{B}\cos\theta_{B}-v_{A}\cos\theta_{A}\right)}^{2}}$$

where Δx and Δy are the differences in the longitude and latitude between the positions of vessels *A* and *B*, respectively, $v_{A}$ and $v_{B}$ are the speeds of vessels *A* and *B*, respectively, and $\theta_{A}$ and $\theta_{B}$ are the courses of vessels *A* and *B*, respectively.

There are three types of near-miss cases based on the relative bearing of vessels based on the International Regulations for Preventing Collisions at Sea 1972 (COLREGs) [42]. In addition, VTS operators must instruct specific avoidance maneuvers in three encounter cases, including head-on, crossing, and overtaking, based on rules 13, 14, and 15 of COLREGs. Therefore, $T_{TOS}$, which is expressed in Equation (7), is calculated by adding the time required to address each encounter situation in a VTS area.

(7) $$T_{TOS}=\overset{H}{\underset{h=1}{\sum }}t_{h}+\overset{C}{\underset{c=1}{\sum }}t_{c}+\overset{O}{\underset{o=1}{\sum }}t_{o}$$

where $t_{h},\;t_{c},$ and $t_{o}$ are the times needed to address head-on, crossing, and overtaking encounter cases, respectively. In addition, *H*, *C*, and *O* are the number of head-on, crossing, and overtaking cases, respectively.

### 3.6. Optimal Number of Workstations

In this study, the unit time of $T_{Task}$ was set to one hour. Therefore, $T_{Task}$ over unit time is the required number of workstations n. WS. The required hourly number of workstations $n.\;WS^{h}$ was calculated as the maximum value to compare the maximum hourly number of workstations $m.\;WS^{h}$ with the current hourly number of operating workstations $c.\;WS^{h}$. For example, suppose that $m.\;WS^{h}$ is less than the $c.\;WS^{h}$, then $c.\;WS^{h}$ is regarded as the minimum number of workstations for the VTS operation. Subsequently, it was divided by 24 to calculate the required number of workstations per day using Equation (8) after summing it up by an hour.

(8) $$n.\;WS=\frac{\sum_{h=1}^{24}max\left(m.\;WS^{h},c.\;WS^{h}\right)}{24}$$

## 4. Case Study

### 4.1. Study Area and Data Preparation

We used the AIS sensor dataset collected for 13 days in June 2014, which was the month with the highest traffic in the year, in the Yeosu VTS area, a harbor in the southern part of the Korean peninsula, to assess the proposed optimal VTS operator staffing level model. Various vessels such as cargo vessels, oil tankers, and containers enter and leave Yeosu VTS, and they cause the highest vessel traffic in Korea. In addition, there are 353 piers and anchorages in the harbor. Moreover, their routes are relatively longer than that of other ports and include various dangerous sections, such as crossing and overtaking. Furthermore, offshore anchorages are used as stopovers for the supply and demand of bunkers. Therefore, the target area is operated as a traffic-safety-specific area.

**Figure 3:**
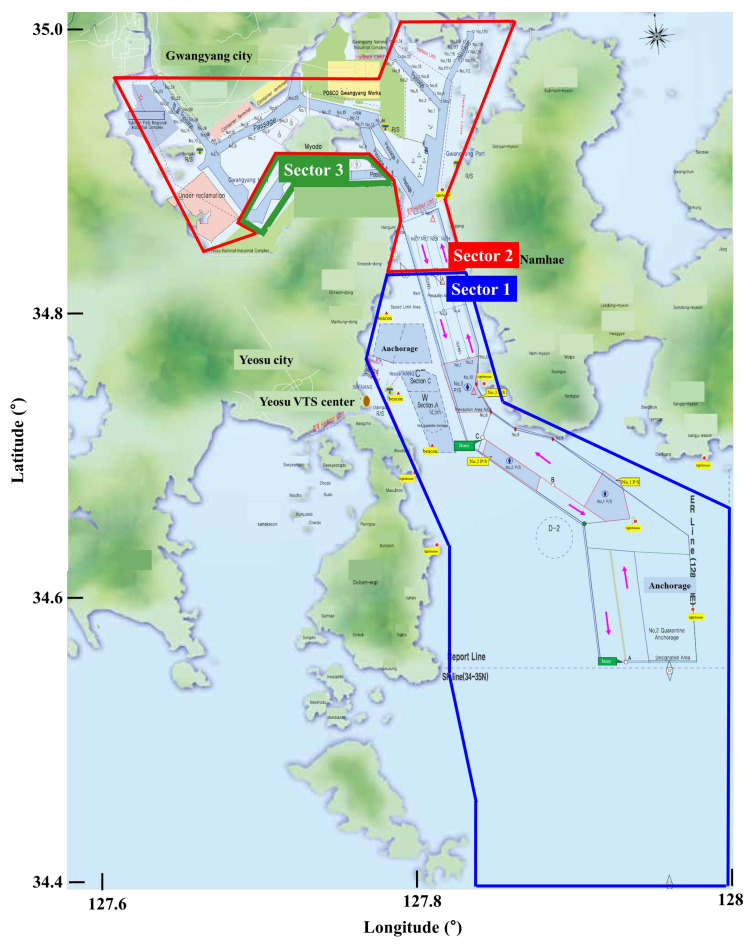
Yeosu VTS area; sector 1 (**blue line**), sector 2 (**red line**), and sector 3 (**green line**).

The Yeosu VTS center is divided into three sectors to ensure intense monitoring. Therefore, there were three workstations in the Yeosu VTS. Figure 3 shows the Yeosu VTS area. Sector 1 covers the traffic separation schemes (TSS), precautionary regions, anchorages, and the open sea. Sector 2 covers the area of the traffic intersections and general cargo terminal including container, cement, steel, and car. Sector 3 covers the area of the dangerous cargo terminals, namely, crude oil, product oil, LPG, and chemical terminals.

Moreover, the vessels should comply with the VTS instruction regarding the anchor position when attempting anchoring in the VTS area and the speed limit regulations. Any vessel navigating in the port should maintain the optimal speed to ensure that anchored or moored vessels are not damaged. The officer of the watch (OOW) of vessels should report advance notice of entry before entering the VTS area. At that time, information, including cargo and current position, is exchanged. All vessels should report their entry, arrival, shifting, 10 min before departure, and departure. Any vessel moving or anchoring in the VTS area should listen to the VTS operation VHF channel 12 or 67 and the emergency channel 16. Vessels that are not subjected to VTS should listen to the VHF channel while navigating in the VTS area to avoid obstructing the course of other vessels.

### 4.2. Required Number of VTS Operators

VTSO operators work shift-wise. The day shift runs from 09:00 to 18:00 h with six operators, followed by the night shift from 18:00 to 09:00 h with seven operators, including one team leader in each shift. During work, the VTS operator performs 1.5 h of monitoring at one workstation. After that, the VTS operators rotate to another workstation 15 m earlier to familiarize themselves with the traffic situation.

Table 1 shows the characteristics of participants via Google Forms. The questionnaire participants were aged between 30 and 50 years. In total, questionnaires from 20 licensed VTS operators, with an average age of 38 years, average merchant vessel officer experience of 3 years, and average VTS experience of 8 years, were collected. The monitoring frequency, time, and weighting factors were obtained from a questionnaire survey. There are four precautionary sections in the Yeosu VTS area that are frequently monitored by VTS operators to prevent vessel collisions. Based on the questionnaire survey, monitoring was performed every 3 min at precautionary sections and once every 10 min in TSS. The vessel complies with the TSS navigating one way. Therefore, the risk of collision in the TSS section between two ships is relatively lower than that in other sections.

Table 2 shows the monitoring weighting factor based on the vessel type and overall length (LOA). The types of ships were classified into five categories: Cargo vessels, tankers, passenger vessels, towing vessels, and small vessels. The cargo vessels include general cargo vessels, bulk carriers, and container vessels, while tankers consist of LNG tankers, LPG tankers, oil tankers, and chemical tankers. It is apparent that the monitoring weighting factor of tankers is more significant than that of cargo vessels, indicating that VTS operators pay more attention to these specific vessels than smaller vessels. The questionnaires on weight factors based on the vessel type and LOA have two parts. Part A was classified into five vessel types: Cargo vessels, tankers, passenger vessels, towing vessels, and small vessels. Part B was classified into four LOA: Less than 75 m, 75–150 m, 151–225 m, and more than 225 m. The questionnaires with a nine-point Likert scale were used as weight factors for influence level by vessel type and LOA at monitoring: 1—not at all influential, 9—extremely influential. Scorings on Likert-based items were converted based on the cargo vessels and LOA 75–150 category. The converted vessel type weight factor and the converted LOA weight factor were multiplied.

The time needed for a sub-item of NAS was obtained by a questionnaire survey on VTS operators, as shown in Table 3. The VTS operators perform tasks consisting of six details if a vessel enters a VTS area. First, the VTS operators communicate with the vessels. Subsequently, they would check the anchorage or wharf, pilot scheduling, and safety information of nearby vessels. Then, the VTS operators would tag a vessel name and symbol on the screen and would be written on a VTS logbook. The communication time includes the readback time. In other cases, the VTS operator enters the harbor management system if a vessel berths, unberths, anchors, or heaves up the anchor. The single-selection question in the seven options was designed to measure the time needed for a detailed task: 0–5 s, 6–10 s, 11–20 s, 21–30 s, 31–40 s, 41–50 s, and 51–60 s. The total average time is a sum of the averages for each sub-item task, which are many respondents’ selections. The total average time needed for a sub-item task is applied in this modeling study. For example, if a vessel enters the VTS area, the operator takes 80.5 s for the tasks of NAS.

**Figure 4:**
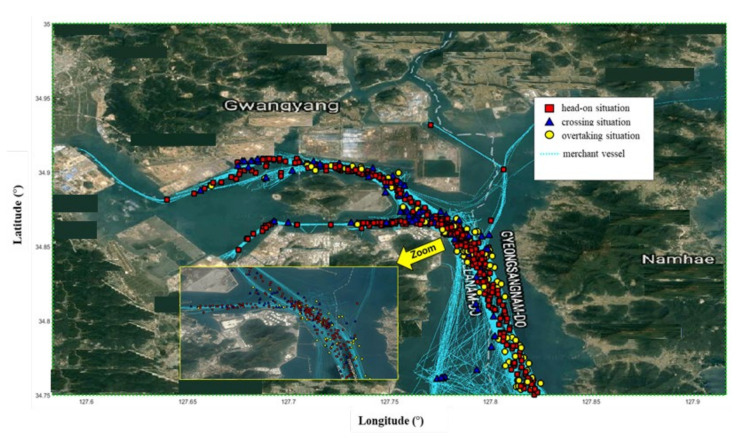
Scatter plot for the near-miss location between merchant vessels in Yeosu VTS area; head-on (**red square**), crossing (**blue triangle**), overtaking situation (**yellow circle**), and merchant vessels trajectories (**cyan dotted line**).

**Figure 5:**
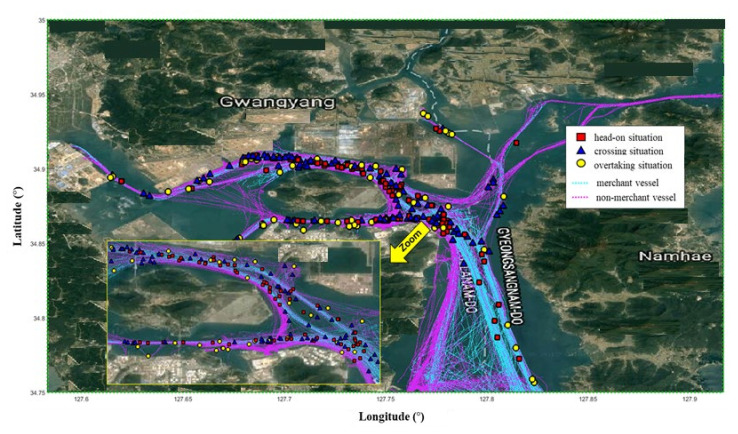
Scatter plot for the near-miss location between merchant vessels and non-merchant vessels in Yeosu VTS area; head-on (**red square**), crossing (**blue triangle**), overtaking situation (**yellow circle**), merchant vessels trajectories (**cyan dotted line**), and non-merchant vessel trajectories (**magenta dotted line**).

Figure 4 shows a scatter plot of near-miss locations between merchant vessels in the Yeosu VTS area. Figure 5 shows the near-miss locations between merchant vessels and non-merchant vessels (i.e., pilot boats, fishing boats, and small boats). The average time needed for TOS was calculated by collecting three days of VHF voice recording data for the same period as the AIS data. Analyzing all 13 days of VHF voice data takes an enormous amount of time, therefore it was judged that there is no need to analyze the entire dataset, so only three days were analyzed. The average time needed for TOS is approximately 30 s from 1320 collected voice files. In the previous study, analyzing VHF voices for three days in the Busan VTS area resulted in an average communication time of TOS of 25 s for 781 cases [43]. This difference of almost 5 s is negligible, so 30 s was considered in our model.

**Figure 6:**
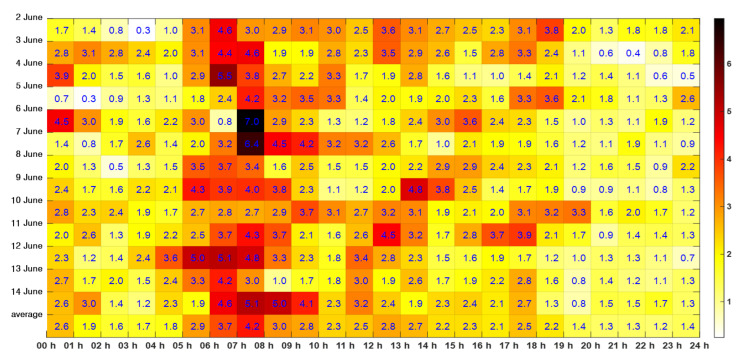
Required hourly number of workstations for Yeosu VTS.

Figure 6 shows the required hourly number of workstations $n.WS^{h}$. Each $n.WS^{h}$ value is the sum of $T_{INS}$, $T_{NAS}$, and $T_{TOS}$. $T_{Task}$ over unit time means the required number of workstations $n.WS^{h}$. For example, the required number of workstations is 1.7 hourly at 00:00–01:00 h on 2 June in Figure 6. The value is the sum of $T_{INS}$ 1.2 h, $T_{NAS}$ 0.3 h, and $T_{TOS}$ 0.2 h. At that time, the VTS operators monitored 78 vessels in the VTS area, provided NAS to 7 vessels, and provided TOS to 22 vessels. The visualization was plotted with a heatmap of the values. It is apparent from the figure that there is much vessel traffic at 05–09 h and relatively little vessel traffic at night. In particular, during rush hours, the required hourly number of workstations $n.WS^{h}$ increases by two to three times compared to the current hourly number of operating workstations $c.\;WS^{h}$.

Table 4 shows the required hourly workstations $n.\;WS^{h}$. The maximum hourly number of workstations $m.\;WS^{h}$ is the maximum value by an hour over 13 days. For example, $m.\;WS^{1}\;\left(4.5\right)$ is the maximum of thirteen values at 00:00–01: 00 h from 2 June to 14 June. The current hourly number of operating workstations $c.\;WS^{h}$ is 3.0 at Yeosu VTS. In addition, the current hourly number is set to the required hourly number of workstations if the maximum hourly number of workstations $m.\;WS^{h}$ is less than the current hourly number of operating workstations $c.\;WS^{h}$. After that, it is divided by 24 to calculate the required number of workstations per day after summing $n.\;WS^{h}$ by hour. In reality, vessel traffic changes dynamically every hour. In addition, it is not easy to predict the vessel traffic in advance and flexibly arrange the VTS operators because the workstation’s position shift time, meals, and rest are fixed. Therefore, it was calculated based on the average required workstations per day. As a result, another workstation should be added to the current workstation at the Yeosu VTS center.

Table 5 shows the stage of calculation for the number of VTS operators per workstation. The number of Yeosu VTS operators per workstation was calculated based on the IALA guidelines [11], concluding that six operators were required per workstation. The number of days for leave of Yeosu VTS operators per year is thirteen, for sickness is three days, and for training or business trips is six days. The standard hours per week are 40. The hours lost, including in breaks and meals, is 2 h per working day.

## 5. Discussion

If the current hourly number of operating workstations $c.\;WS^{h}$ is less than the required number of workstations n. WS derived from the proposed method, additional workstations are required. Thus, the current VTS operators are overloaded at specific rush hours. This leads to a problem that the current VTS operator may not prevent marine accidents due to failure to provide timely safety information. In addition, in the event of a marine accident during traffic congestion, the time to recognize the situation for the VTS operator may be long enough to delay the initial response [8,9].

According to Figure 6, we can distinguish between rush hour and non-rush hour. Approximately, the rush hour zone is 05–09 h. However, the rush hour zone may vary from day to day, depending on vessel traffic. For example, it required 4.5 workstations at 00–01 h 6 June, but only 0.8 workstations at 06–07 h on the same day. It is not easy to dynamically arrange the VTS operators according to the traffic situation since mealtimes, break times, and workstation shift times are set for each operator. If the operators are dynamically deployed to the workstation, the operators will be exposed to more stress due to irregular times. Moreover, dynamically deploying only one VTS operator with less experience during non-rush hours may result in insufficient initial response to marine accidents. Therefore, developing a model for appropriately and dynamically deploying VTS operators considering the rush hour and individual capabilities is left for future research.

To compare the validity of the proposed method, we calculated the number of workstations of Yeosu VTS based on the Xie model [1]. The comparative model derived the number of workstations based on 2014 annual statistics. Table 6 shows the time needed for each item based on the annual statistics. The number of entering and leaving vessels was 29,582 and 29,656 in 2014, respectively. Moreover, 4050 vessels passed through the VTS area. The distance from the south VTS reporting line to the berth is approximately 38 nm, and the distance from the west to the east VTS reporting line is 9 nm. The parameters were applied with target identification for 20 s, sail planning for 30 s, and broadcasting safety information for 60 s based on the Xie model [1]. The required number of workstations n. WS based on the statistic-based macroscopic model was 3.0, which is lower than the results of the proposed microscopic model.

The questionnaire-based survey for 20 Yeosu VTS operators was conducted to verify the proposed method. More than 95% of VTS operators responded that the current hourly number of operating workstations is short, and more workstations are needed. They responded that it was well reflected in the proposed method considering TOS because the VTS operators address dangerous vessel near-miss cases every hour. Seventy percent of the respondents said the annual statistics were unreliable. It was found that this is because it may be omitted from statistical data calculated by hand. This case study confirmed that an appropriate number of VTS workstations could be derived based on AIS data, even without annual statistical data.

## 6. Conclusions

VTS can potentially reduce maritime accidents. In addition, having sufficient operating personnel is its most critical component. The VTS objective cannot be achieved if services are not provided on time, which causes workload and stress due to the insufficient number of VTS operators.

In this study, we developed a new, microscopic method for calculating the number of optimal VTS operators from AIS data, a questionnaire survey, and a VHF voice analysis. Particularly, this study helps to quantitatively measure the number of workstations required at a VTS center.

The proposed microscopic method proposed an advantage. It does not depend on a statistic-based macroscopic model for calculating the number of VTS operators. Instead, it uses observable AIS sensor data and VHF voice data, which are easily collected at the VTS center. The macroscopic model-based calculations of the number of VTS operators require annual statistical vessel traffic, which cannot be calculated using an automated programming module but are obtained manually, resulting in low reliability. It is expected that VTS operator fatigue and workload can be reduced, helping prevent vessel accidents by providing timely services even though the vessel traffic is congested if they are deployed to a sufficient number of workstations.

There remain opportunities for further studies to improve upon the proposed method. In the future, it is necessary to improve the parameters derived from the questionnaire using the VTS operators’ eye-tracking method. Furthermore, by deploying a dynamically flexible number of operators that reflect the vessel traffic rush hour, the problem of setting the sector area to be monitored by each workstation should be solved. A sector-based near-miss density map is also needed to help set reasonable monitoring times for each sector.

## Figures and Tables

**Table 1:** Questionnaire participants characteristics.

Characteristics		Number	%
Gender	Male	18	90
	Female	2	10
Age	31–40 years	12	60
	41–50 years	1	5
	>51 years	7	35
Merchant vessel officer experience	<3 years	6	30
	3–5 years	12	60
	6–10 years	2	10
VTS experience	<2 years	6	30
	2–5 years	2	10
	6–10 years	5	25
	>10 years	7	35

**Table 2:** Monitoring weighting factor based on the vessel type and length.

Type	LOA	Monitoring Weighting Factor
Cargo vessels	<75 m	0.8
	75–150 m	1.0
	151–225 m	1.3
	>225 m	1.6
Tankers	<75 m	1.0
	75–150 m	1.4
	151–225 m	1.8
	>225 m	2.2
Passenger vessels	<75 m	0.9
	75–150 m	1.1
	151–225 m	1.5
	>225 m	1.8
Towing vessels	<75 m	1.0
	75–150 m	1.3
	151–225 m	1.7
	>225 m	2.1
Small vessels	<75 m	0.2

**Table 3:** Detailed task of a VTS operator and total time needed for sub-item tasks of NAS.

Sub-Item Task	Detailed Task of a VTS Operator	Time Needed for a Detailed Task	Total Average Time Needed for a Sub-Item Task
$t_{e}$ *	Communication with vessels by VHF	21–30 s	80.5 s
	Checking anchorage or wharf information	11–20 s	
	Checking pilot scheduling	6–10 s	
	Checking of safety situation of nearby vessels	11–20 s	
	Tagging vessel name and symbol	6–10s	
	Filling in VTS logbook	6–10 s	
$t_{l}$ *	Communication with vessels by VHF	11–20 s	23.5 s
	Filling in VTS logbook	6–10 s	
$t_{a}$ *	Communication with vessels by VHF	11–20 s	39.0 s
	Entering information into the harbor management system	11–20 s	
	Filling in logbook	6–10 s	
$t_{h}$ *	Communication with vessels by VHF	21–30 s	80.0 s
	Entering information into the harbor management system	11–20 s	
	Checking wharf information	11–20 s	
	Checking of safety situation of nearby vessels	11–20 s	
	Filling in logbook	6–10 s	
$t_{p}$ *	Communication with vessels by VHF	11–20 s	39.0 s
	Filling in logbook	6–10 s	
	Adjustment order of pilot boarding and disembarkation	11–20 s	

* $t_{e}$: Times needed to communicate with the *e*th entering vessel in the VTS area; $t_{l}$: The *l*th leaving vessel; $t_{a}$: The *a*th anchoring or berthing vessel; $\;t_{h}$: The *h*th heaving up anchor vessel or unberthing; $t_{p}$: The *p*th vessel with a pilot on board.

**Table 4:** Required hourly number of workstations ($n.\;WS^{h}$) for Yeosu VTS.

Hours	$m.\;WS^{h}\;$	$c.\;WS^{h}\;$	$n.\;WS^{h}\;$
00–01	4.5	3.0	4.5
01–02	3.1	3.0	3.1
02–03	2.8	3.0	3.0
03–04	2.6	3.0	3.0
04–05	3.6	3.0	3.6
05–06	5.0	3.0	5.0
06–07	5.5	3.0	5.5
07–08	7.0	3.0	7.0
08–09	5.0	3.0	5.0
09–10	4.2	3.0	4.2
10–11	3.3	3.0	3.3
11–12	5.1	3.0	5.1
12–13	4.9	3.0	4.9
13–14	4.8	3.0	4.8
14–15	3.8	3.0	3.8
15–16	3.6	3.0	3.6
16–17	3.7	3.0	3.7
17–18	3.9	3.0	3.9
18–19	3.8	3.0	3.8
19–20	3.3	3.0	3.3
20–21	1.8	3.0	3.0
21–22	2.0	3.0	3.0
22–23	1.9	3.0	3.0
23–24	2.6	3.0	3.0
n. WS			4.0

**Table 5:** Calculation for the number of VTS operators per workstation.

Stage	Calculation
Stage 1: Actual hours per year	8766 h = Hours per day (24 h) × Actual days per year (365.25 d)
Stage 2: Hours after deductions	1911.1 h = Hours before deductions per year (40 h × 365.25 d/7 d)—Hours for leave, sickness, and training per year {8 h × (13 d + 3 d + 6 d)}
Stage 3: Hours lost per year	477.7 h = Working days per year (1911.1 h/8 h) × Hours lost (break, meal) per working day (2 h)
Stage 4: Total duty hours per year	1433.3 h = Hours after deductions (1911.1 h)—Hours lost (break, meal) per year (477.7 h)
Stage 5: Number of VTS operators per workstation	6.1 = Actual hours per year (8766 h)/Total duty hours per year (1433.3 h)

**Table 6:** Comparative experiment of the number of workstations of Yeosu VTS based on the Xie model [1].

Item	Calculation	Hour
Time needed for target identification and label	{(29,582 + 29,656 + 4050) × 20 s}/(365 × 3600 s)	1.0 h
Time needed for replying shipping report	{(29,582 + 29,656 + 4050) × 20 s}/(365 × 3600 s)	1.0 h
Time needed for tracking monitoring	{(29,582 + 29,656) × 0.1 × 38 nm}/(365 × 10 kt) (4050) × 0.1 × 9 nm}/12 kt	62.5 h
Time needed for broadcasting safety information	(137,713 × 60 s)/(365 × 3600 s)	6.3 h
Time needed for sail planning	(20,234 × 30 s)/(365 × 3600 s)	0.5 h
Other business work time	(15,370 × 60 s)/(365 × 3600 s)	0.7 h
Total		71.9 h
n. WS		3.0

## Data Availability

Not applicable.
